# Malarial Hepatopathy in Children Visiting a Tertiary Healthcare Hospital in Karachi

**DOI:** 10.7759/cureus.6696

**Published:** 2020-01-18

**Authors:** Nathumal Maheshwari, Mehmood Shaikh, Rewa Chand, Hemandas Maheshwari, Mehrunnisa Yasir

**Affiliations:** 1 Pediatrics, Shaheed Mohtarma Benazir Bhutto Medical College, Karachi, PAK; 2 Neonatal Intensive Care Unit, Jinnah Sindh Medical University, Karachi, PAK; 3 Pediatric Surgery, Shaheed Mohtarma Benazir Bhutto Medical College, Karachi, PAK; 4 Pediatrics, St. Richard's Hospital, Chichester, GBR; 5 Medical Intensive Care Unit, National Institute of Child Health, Karachi, PAK

**Keywords:** plasmodium falciparum, plasmodium vivax, malarial hepatopathy

## Abstract

Objective

To determine the frequency of malarial hepatopathy in children that are visiting Lyari General Hospital in Karachi, Pakistan.

Study design

Cross sectional descriptive study.

Material and methods

Patients with age between two months and 15 years, who had positive blood film for Plasmodium falciparum or P. vivax, were included in the study. All patients were monitored for malarial hepatopathy.

Result

A total of 241 cases were included in the study. Mean age at admission was 4.1 ± 1.3 years and male to female ratio was 1.2:1. There were 133 (55.2%) cases of P. vivax, while 108 (44.8%) were of P. falciparum. Malarial hepatopathy was observed in 37 patients (15.4%). Malaria hepatopathy was present in 24.1% and 8.3% children having P. falciparum and P. vivax, respectively. Malaria hepatopathy was present in 24%, 18% and 6% in age groups two months to five years, >5 years to 10 years and >10 years, respectively.

Conclusion

Malarial hepatopathy was observed in about one-sixth of study population and it was more common between two months and five years age group.

## Introduction

Malaria is one of the leading causes of morbidity and mortality in children worldwide [1]. Malaria is generally caused by four Plasmodium species (falciparum, malariae, vivax and ovale). In the islands of Borneo and peninsular Malaysia malaria occurred due to fifth species, Plasmodium knowlesi but the most dangerous species are P. falciparum and P. vivax [2,3].

Malaria is the second most frequently observed disease in under-developed countries [4]. It is endemic in more than one hundred countries, predominately in Africa, Asia, Central America, and South America with about 40% of the world’s population at risk [5-8]. P. falciparum and P. vivax are endemic in Pakistan [9]. Agricultural practice, irrigation network, and monsoon rains are major factors to enhance the malarial potential in many areas [4].

According to the World Health Organization (WHO), in 2015 there were an estimated 429,000 deaths worldwide due to malaria, of which 99% were due to P. falciparum alone. Seventy percent (approximately 303,000) of the global estimate were of children under the age of five years [10].

Malaria in children can mimic many diseases and there are no absolute clinical features [11]. It may present with high grade fever with chills, headache, sweating, myalgia, abdominal pain, vomiting, diarrhea, anemia, hepatomegaly, splenomegaly and jaundice [12]. In endemic regions, malaria can present atypically due to development of immunity, increasing resistance to antimalarial drugs, and the indiscriminate use of antimalarial drugs [1]. The most common complications of malaria are disseminated intravascular coagulation (DIC), hepatopathy, cerebral malaria, acute kidney injury, hypoglycemia and severe anemia [13, 14]. Hepatic dysfunction is common in either isolated P. falciparum or a mixed infection with P. vivax [15]. Jaundice in malaria is due to intravascular hemolysis of parasitized erythrocytes, hepatic dysfunction and DIC [16]. Severe jaundice is reported from many countries of South-east Asia, but according to the WHO the signs of gross hepatocyte dysfunction and hepatic encephalopathy do not occur in these patients [11]. Malaria hepatitis is described by hyperbilirubinemia (> 3 mg/dl), elevated transaminase more than three folds the normal levels and in the absence of clinical or serological evidence of viral hepatitis [17].

There is a paucity of data related to malarial hepatopathy worldwide. Hence, we decided to conduct this study at our tertiary care center.

## Materials and methods

This was a cross sectional descriptive study conducted at Pediatrics Department, Shaheed Mohtarma Benazir Bhutto Medical College, Lyari Karachi and Lyari General Hospital, Karachi for a period of six months from May to October 2018. The study was initiated after approval from the Institutional Review Board and obtaining informed consent from the parents/guardian. Patients with age between two months to 15 years, who had positive blood film for Plasmodium falciparum or P. vivax, were included in the study by consecutive non-probability sampling, until the desired number of patients was completed. Criteria used to diagnose malarial hepatopathy were demonstration of Plasmodium infection: falciparum/vivax, with serum bilirubin >3 mg/dl and at least two to three-fold rise in transaminases enzymes (alanine transaminase, aspartate aminotransferase) and normal or abnormal prothrombin time (PT). Patients with evidence of liver disease or enteric hepatitis were excluded from the study. Data including history and examination were collected on specially designed proforma. Investigations included complete blood counts, peripheral blood film for detailed morphology of RBCs, thick and thin blood films for malarial parasites, prothrombin time, total serum bilirubin, conjugated and unconjugated bilirubin, and serum aspartate transaminase (AST) and alanine transaminase (ALT) levels. Results were analyzed using SPSS version 20 (IBM Corp., Armonk, NY). Qualitative variable was presented as frequency and percentages, while quantitative variables presented in mean and standard deviation.

## Results

**Figure 1:**
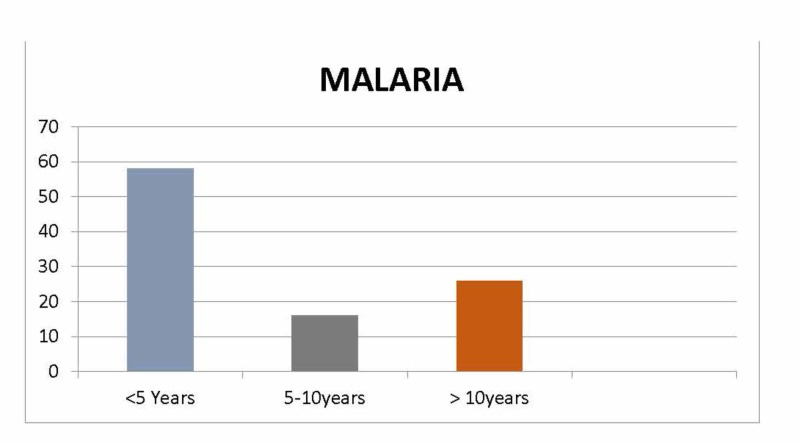
Age of the patients with malaria.

A total of 241 cases of blood smear positive for Plasmodium falciparum/vivax were included in the study. Out of 241 patients, 130 were males (53.9%) and 111 were females (46.1%) resulting in male:female ratio of 1.2:1. Mean age at admission was 4.1 ± 1.3 years (ranged from 0.5-13 years). There were 133 (55.2%) cases of P. vivax, while 108 (44.8%) of P. falciparum. Mean serum bilirubin level, serum alanine transferase (ALT) and aspartate amino transferase (AST) were 1.8 ± 4.5 mg/dl ± SD (Range: 0.2-26 mg/dl), 40.1 ± 46.2 IU/L ± SD (Range: 9-165 IU/L) and 35.6 ± 41.3 IU/L ± SD (Range: 10-189 IU/L), respectively. Abnormal PT was seen only in 27 (11.2%) children. Majority (140; 58%) of children were aged between two months and five years (Figure 1).

**Figure 2:**
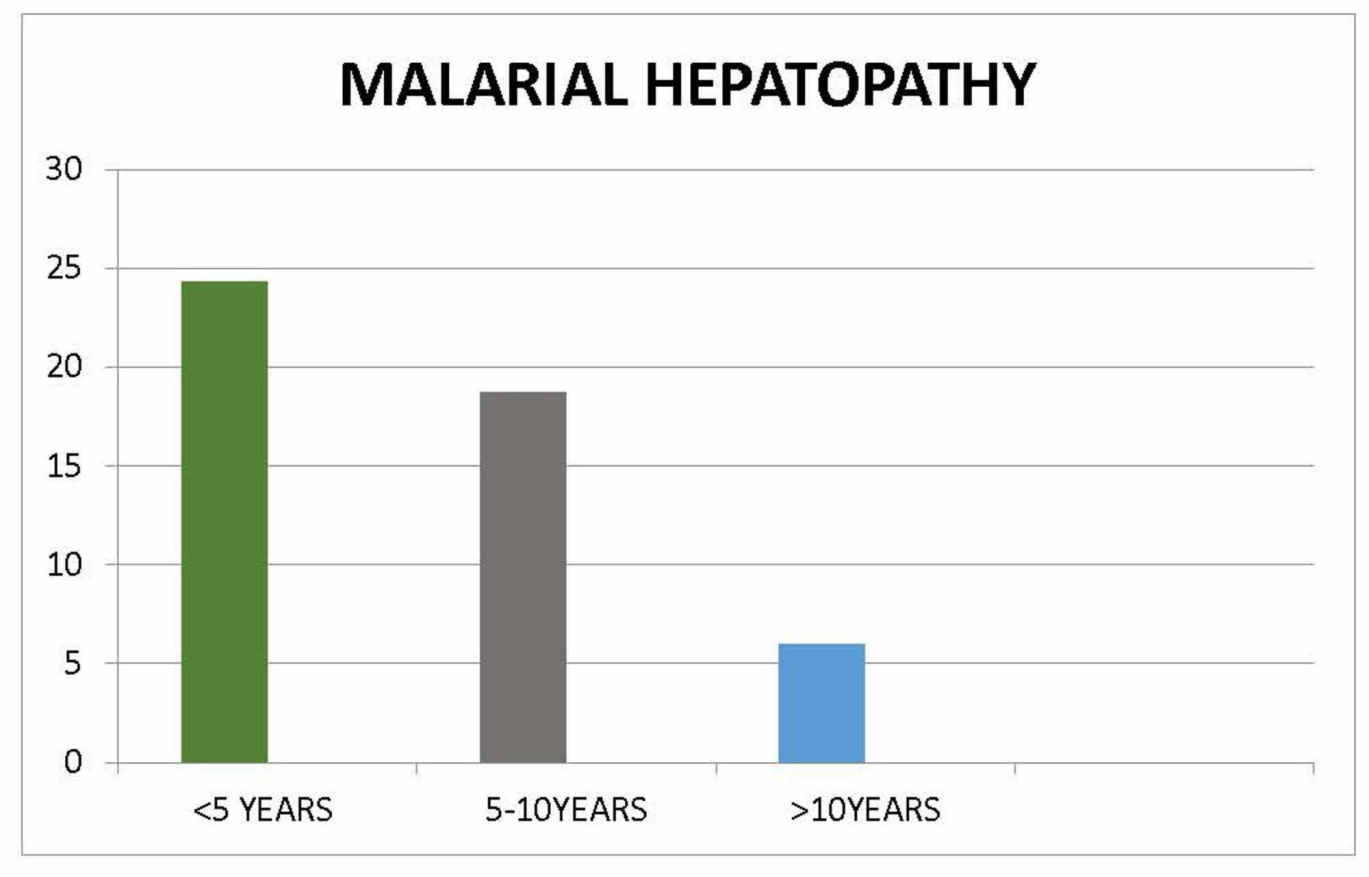
Age of the patients with malaria hepatopathy.

In 37 patients (15.4%), serum transaminase level was more than thrice the upper limit of normal levels. There were 22 males (59.4%) and 15 females (40.6%), with male:female of 1.46:1. Mean age of children with malarial hepatopathy was 3.1 ± 1.1 years with a range of 0.5-12.5 years. Majority (9; 24.2%) of children had age between two months and five years (Figure 2).

Mean serum bilirubin level, serum ALT and AST in malarial hepatopathy patients were 13.1 ± 9.3 mg/dl ± SD (Range: 12-26 mg/dl), 125.2 ± 8.2 IU/L ± SD (Range: 120-165 IU/L) and 116.2 ± 6.3 IU/L ± SD (Range: 110-189 IU/L), respectively. Malarial hepatopathy with respect to the type of Plasmodium is shown in Table 1.

**Table 1:** Malarial hepatopathy with respect to type of plasmodium.

Type	Total	Malarial Hepatopathy	Percent
Plasmodium vivax	133	11	8.3
Plasmodium falciparum	108	26	24.1

## Discussion

Malaria remains a serious health problem worldwide, which threatens millions of people and inflicts a huge burden in terms of complications and mortality [18-22]. Jaundice is one of the severe manifestations of malaria. It may present alone or with other complication of malaria and vary in intensity. The spectrum of presentation ranges from mild jaundice due to the hemolysis to severe jaundice due to hepatic dysfunction [23].

Out of 241 patients, 130 were males (53.9%) and 111 were females (46.1%) resulting in male:female ratio of 1.2:1, which is consistent with Hussain et al., Kochar et al. and Sahar et al. [4,24,25]. Male predominance may be due to more outdoor activities as compared to female.

In our study, frequency of P. vivax was more common than P. falciparum which is consistent with other studies done by Hussain et al. and Nadeem et al. [4, 26]. However, it was contrary to another study by Khadim and Akbar [27, 28].

Hepatic dysfunction in children was found to be 15.35% which was contrary to other study by Mannu et al. [29]. P. falciparum showed hepatopathy in 32.2% of cases in Indian study done by Satpathy et al. [30]. The results are conformity with our study where hepatopathy due to P. falciparum was in 24.1% cases. In comparison to study by Kochar et al., which showed that hepatic hepatopathy was present in 44.3% (35/79), 26.2% (17/65) and 16.7% (1/6) children having P. falciparum, P. vivax and mixed infections, respectively [24]. Our study revealed malaria hepatopathy was present in 24.1% and 8.3% children having P. falciparum and P. vivax, respectively. In a study conducted by Kochar et al., it was described that hepatic hepatopathy was present in 24.7%, 17.9% and 6.6% in the age groups of two months to five years, >5 years to 10 years and >10 years, respectively [24]. The results are in conformity with our study.

## Conclusions

Malarial hepatopathy was observed in about one-sixth of study population and interestingly it was found to be more common in children between two months and five years. We, therefore, conclude that screening for hepatopathy should be mandatory in patients that have P. falciparum and P. vivax infections. Further studies with large sample sizes are required to fully understand malarial hepatopathy in depth.
